# The impact of maternal genetic merit and country of origin on ewe reproductive performance, lambing performance, and ewe survival

**DOI:** 10.1093/tas/txab070

**Published:** 2021-04-21

**Authors:** Nicola Fetherstone, Nóirín McHugh, Tommy M Boland, Fiona M McGovern

**Affiliations:** 1 Teagasc, Animal and Grassland Research and Innovation Centre, Mellows Campus, Athenry, Co. Galway H65 R718, Ireland; 2 School of Agricultural Science, University College Dublin, Belfield, Dublin 4, D04 V1W8, Ireland; 3 Teagasc, Animal and Grassland Research and Innovation Centre, Moorepark, Fermoy, Co. Cork, P61 C996, Ireland

**Keywords:** breeding, genetics, index, production, sheep

## Abstract

The objective of this study was to investigate the impact of the ewe’s maternal genetic merit and country of origin [New Zealand (**NZ**) or Ireland] on ewe reproductive, lambing, and productivity traits. The study was performed over a 4-yr period (2016–2019) and consisted of three genetic groups: high maternal genetic merit (NZ), high maternal genetic merit Irish (High Irish), and low maternal genetic merit Irish (Low Irish) ewes. Each group contained 30 Suffolk and 30 Texel ewes, selected based on the respective national maternal genetic indexes; i.e., either the NZ Maternal Worth (NZ group) or the €uro-star Replacement index (Irish groups). The impact of maternal genetic merit on reproductive traits such as litter size; lambing traits such as gestation length, birth weight, lambing difficulty, mothering ability; and productivity traits such as the number of lambs born and weaned was analyzed using linear mixed models. For binary traits, the impact of maternal genetic merit on reproductive traits such as conception to first artificial insemination (**AI**) service; lambing traits such as dystocia and perinatal lamb mortality; and productivity traits such as ewe survival was analyzed using logistic regression. NZ ewes outperformed Low Irish ewes for conception to first AI (*P* < 0.05) and litter size (*P* = 0.05). Irish ewes were more likely to suffer from dystocia [6.84 (High Irish) and 8.25 (Low Irish) times] compared to NZ ewes (*P* < 0.001); birth weight and perinatal mortality did not differ between groups (*P* > 0.05). Lambs born from NZ ewes were 4.67 [95% confidence interval (**CI**): 1.89–11.55; *P* < 0.001] and 6.54 (95% CI: 2.56–16.71; *P*<0.001) times more likely to stand up and suckle unassisted relative to lambs born from High or Low Irish ewes, respectively. NZ and High Irish ewes had a greater number of lambs born and weaned throughout the duration of the study compared to their Low Irish counterparts (*P*<0.001). NZ ewes tended to be more likely to survive from one year to the next compared with Low Irish ewes (*P*=0.07). Irish ewes of high maternal genetic merit outperformed their low counterparts in total number of lambs born and weaned per ewe, but performance did not differ across other traits investigated. This highlights the importance of continuous development of the Irish maternal sheep index to ensure favorable improvements in reproductive, lambing, and productivity traits at the farm level. Overall, results demonstrate the suitability of NZ genetics in an Irish production system.

## IMPLICATIONS

The widespread use of animals of high maternal genetic merit, regardless of their country of origin, may positively impact ewe reproductive, lambing, and productivity performance, therefore having the potential to increase national productivity, efficiency, and profitability if the use of high maternal genetic merit animals was implemented through a widespread national breeding program.

## INTRODUCTION

Genetic indexes provide producers with a valuable tool to make more informed breeding decisions which can result in increased farm productivity and profitability as demonstrated within the Irish dairy industry (Ramsbottom et al., 2012). However, validation of genetic indexes is vitally important to increase farmers’ confidence in breeding programs as well as demonstrating that genetic progress is achievable. Controlled experiments of animals of divergent genetic merit across both cattle (McCabe et al., 2017; O’Sullivan et al., 2020) and sheep (Lewis et al., 1996; Marquez et al., 2013) have shown that genetic evaluations are reflected in differences in phenotypic animal performance. However, for sheep studies, a greater focus has been placed on terminal traits rather than maternal traits. To date, the national maternal sheep breeding objective in Ireland, the €uro-star Replacement index (Bohan et al., 2019), has not been validated against other international evaluation systems.

The New Zealand (NZ) sheep production system is broadly similar to that of Ireland, with both operating a predominately grass-based, seasonal, and export-focussed system, albeit with NZ operating a more extensive system. The similarities in both production systems are reflected in both the traits and the relative emphasis placed on these traits included in both the NZ and Irish maternal genetic indexes (Santos et al., 2015). However, as reported by Santos et al. (2015), the genetic progress in the Irish maternal sheep index has been slow (€0.28/lamb per year) compared with the corresponding gains reported in NZ (€1.16/lamb per year). To date, however, Irish and NZ sheep of high maternal genetic merit have not been compared in a common environment. The objective of the study was to investigate the effect of maternal genetic merit on ewe reproductive, lambing, and productivity traits across animals divergent in their maternal genetic merit from two countries of origin, NZ and Ireland.

## MATERIALS AND METHODS

### Study Design

This study was performed over a 4-yr period (2016–2019) at Teagasc, Animal and Grassland Research Centre, Mellows Campus, Athenry, Co. Galway, Ireland (53.288024 latitude, 8.778380 longitude). All procedures were conducted under approval from the Teagasc Animal Ethics Committee on Experimental Animal Use (TAEC56-2014) and the Health Protection Regulation Authority (AE19132 / P039) in accordance with the Cruelty to Animals Act 1876 and the European Communities Regulations, 1994.

Three genetic groups of ewes, balanced for breed and age, were established, containing 60 NZ ewes of high maternal genetic merit, 60 Irish ewes of high maternal genetic merit (High Irish), and 60 Irish ewes of low maternal genetic merit (Low Irish). A cohort of NZ animals selected based on the New Zealand Maternal Worth Index was imported to Ireland in 2013 and 2014 ahead of the commencement of this study with mating in October 2015; these animals represented those ranked within the top 40% across breed for maternal genetic merit (Byrne et al., 2012) and were selected from six progressive flocks that achieved the equivalent genetic gain of €0.18 under the New Zealand Maternal Worth Index as previously discussed by Fetherstone et al. (2021). Irish ewes were selected based on their genetic merit at the time of entry into the study for the Irish maternal genetic index for sheep; i.e., the €uro-star Replacement index (Bohan et al., 2019). The High Irish and Low Irish ewes represented the top and bottom 20% of animals for maternal genetic merit within their breed, respectively. Each genetic group consisted of 30 purebred Suffolk and 30 purebred Texel ewes. All ewes remained in their allocated genetic group for the duration of their productive life. Any ewe that was removed from the genetic group during the study due to health issues or ewe mortality was immediately replaced by another ewe that was of similar genetic merit, breed, parity, rearing type, and litter size. In total, 350 ewes formed part of the trial across the 4-yr period. The average maternal genetic merit value, i.e., the Irish €uro-star Replacement index, at the start of the study was €0.06 ± 0.741, €1.04 ± 0.617, and –€0.68 ± 0.729 for the NZ, High Irish, and Low Irish genetic groups, respectively.

### Experimental Design

In autumn of each year, each ewe (experimental unit) was estrus synchronized and mated via laparoscopic artificial insemination (**AI**). The total cohort of ewes was randomly divided into two groups, balanced for maternal genetic merit, breed, and age, and inseminated 1 wk apart. All ewes were bred within the maternal genetic merit group and within breed. Rams were introduced to ewes for two repeat cycles following AI, where all trial ewes were mated within 21 d from the day of AI. In early December each year, ewes were housed indoors. Winter shearing was carried out at housing and all ewes were ultrasound pregnancy scanned between 80 and 90 d post AI. All ewes received grass silage *ad libitum*, while concentrate supplementation was provided based on silage quality and ewe energy requirements according to litter size (Alderman and Cottrill, 1996) from 8 wk prior to the predicted lambing date. Lambing commenced in the last week of February each year. Postpartum, ewes were individually penned for 48 h to encourage ewe-lamb bonding; thereafter, ewes and lambs were turned out onto a perennial ryegrass (*Lolium perenne*) and white clover (*Trifolium repens*) sward at a stocking rate of 12 ewes per ha. A rotational grazing system was operated where target pregrazing heights range from 7 to 9 cm. Postgrazing heights were 3.1 cm for the first rotation and 4.1 cm thereafter. The maximum number of lambs reared per ewe was 2; for ewes with a litter size of ≥3, excess lambs were removed and placed into an artificial rearing unit or cross-fostered onto another ewe within the same genetic merit group. All genetic merit groups grazed separate farmlets throughout the grazing season.

### Description of Critical Methods

#### Reproductive performance.

Conception rate to first AI service (binary trait) was defined as whether or not a ewe was confirmed in lamb to AI. Ewe barren rate (binary trait) was defined as whether or not a ewe was pregnancy scanned in lamb after AI and two repeat cycles. Pregnancy scan rate was defined as the number of fetuses scanned per ewe including barren ewes. Litter size was defined as the number of lambs born per ewe excluding barren ewes. Ewe survival (binary trait) was defined as whether or not a ewe that was bred in 1 yr was retained for breeding the following year.

#### Lambing performance.

Within the first 24 h postpartum, all lambs were weighed using a portable weighing scale, sexed, tagged, and linked to their genetic dam; litter size and date and time of birth were also recorded. Gestation length (days) was calculated as the difference between the date of conception and the date of lambing. Lambing difficulty was scored on a scale of 1–4, where 1= lambed without assistance, 2= slight assistance, 3= manual delivery, and 4= considerable difficulty or veterinary assistance. Lambing difficulty was also dichotomized into lambing dystocia (binary trait) whereby ewes with considerable difficulty or veterinary assistance were coded separately to all other scores. Litter vigor was recorded on a two-point scale (Annett et al., 2012), in which 1 = the litter required no assistance to suckle and 2 = the litter required assistance to suckle. Mothering ability was scored on how easily the ewe followed the lamb(s), using a scale of 1–3 (Earle et al., 2017), in which 1 = always follows lambs, 2 = stands well back from lambs, and 3 = leaves lambs. Perinatal lamb mortality was defined as whether or not the lamb survived for the first 24 h after birth.

#### Productivity.

The total number of lambs born per ewe (alive and dead) and weaned (~100 d postpartum) per ewe over the duration of the study (4 yr) was calculated as an approximation for the ewe’s productive life.

### The Statistical Analysis of the Results

The effect of maternal genetic merit (NZ, High Irish, or Low Irish) on reproduction traits (pregnancy scan and litter size), lambing traits (gestation length, lambing difficulty, mothering ability, and birth weight) and ewe productivity traits (total number of lambs born and weaned) was analyzed using a linear mixed model in PROC Mixed (SAS Inst. Inc., Cary, NC), with maternal genetic merit, ewe breed, ewe parity, sire of the lamb, date or week of trait measurement included as fixed effects. For lambing traits, birth type and sex of the lamb were also included as fixed effects where appropriate. For ewe productivity traits, maternal genetic merit, ewe breed, sire of the lamb, and the ewe’s total number of lambing events over the duration of the study were included as fixed effects. Across all traits, sire of the ewe was included as a random effect and year was included as a repeated effect where appropriate.

For the binary traits of conception to first AI service, barren rate, ewe survival, lamb dystocia, litter vigor and perinatal lamb mortality; the log of the odds were modeled using logistic regression in PROC GENMOD (SAS Inst. Inc., Cary, NC), with maternal genetic merit, ewe breed, ewe parity, and date or week and year of trait measurement included as fixed effects. For lambing traits and for ewe survival, birth type was also included as a fixed effect. Birth weight and sex of the lamb were included as fixed effects for perinatal lamb mortality. Across all binary traits, odds ratios were calculated as the exponent of the model solutions.

## RESULTS

### Reproductive Performance

Conception rates to first AI service presented as raw means were 82%, 80%, and 74% for NZ, High Irish, and Low Irish, respectively. NZ ewes were 2.86 times (95% CI: 1.22–6.73; *P*<0.05) more likely to hold first AI service compared with Low Irish ewes (Table 1). High Irish did not differ from either NZ or Low Irish ewes (*P*>0.05). Barren rates after AI and two repeat cycles did not differ (*P*>0.05) and averaged 7.1%, 6.8%, and 7.8% for NZ, High Irish, and Low Irish, respectively. Overall pregnancy scan rates did not differ between the three genetic groups (*P*>0.05, Table 2). However, ewes within the NZ genetic group had a greater litter size than the Low Irish ewes (*P*=0.05, Table 2). Across the 4 experimental years, ewes were present for an average of 2.5 lambing events. Ewes of NZ origin tended to be more likely to survive in the flock from one year to the next when compared with Low Irish ewes (*P*=0.07, Table 1).

**Table 1.** OR (95% CI in parentheses) with associated *P*-value for annual reproductive and lambing traits by ewe maternal genetic merit* (NZ, High Irish, Low Irish)

Variable	Class contrast	OR (95% CI)	*P*-value
Reproductive traits			
Conception to first AI service, per year	NZ vs. High Irish	1.66 (0.67–4.08)	NS
	NZ vs. Low Irish	2.86 (1.22–6.73)	0.01
	High Irish vs. Low Irish	1.73 (0.73–4.08)	NS
Ewe survival, per year	NZ vs. High Irish	1.78 (0.75–4.24)	NS
	NZ vs. Low Irish	2.24 (0.94–5.37)	0.07
	High Irish vs. Low Irish	1.23 (0.54–2.94)	NS
Lambing traits			
Dystocia, per lambing	High Irish vs. NZ	6.84 (2.28–20.54)	<0.001
	Low Irish vs. NZ	8.25 (2.75–24.73)	<0.001
	Low Irish vs. High Irish	1.21 (0.49–2.98)	NS
Litter vigor, per lambing	NZ vs. High Irish	4.67 (1.89–11.55)	<0.001
	NZ vs. Low Irish	6.54 (2.56–16.71)	<0.001
	High Irish vs. Low Irish	1.40 (0.52–3.75)	NS

NS, not significant (*P* > 0.05).

*The reference category was the first class listed.

**Table 2.** The effect of ewe maternal genetic merit (NZ, High Irish, Low Irish) on annual reproductive and lambing performance traits (least square means and standard error in parenthesis)

	Ewe maternal genetic merit			
	NZ	High Irish	Low Irish	*P*-value
Reproductive traits				
Pregnancy scan rate, lambs scanned per ewe per year	1.84 (0.048)	1.62 (0.049)	1.59 (0.049)	NS
Litter size, lambs born per ewe lambing	1.96^*a*^ (0.050)	1.76^*a*,*b*^ (0.049)	1.73^*b*^ (0.50)	0.05
Lambing traits				
Gestation length, d	147. 9 (0.20)	148.2 (0.20)	148.4 (0.20)	NS
Birth weight, kg	5.11 (0.084)	5.27 (0.080)	5.10 (0.082)	NS
Lambing difficulty	2.09 (0.083)	2.61 (0.077)	2.65 (0.081)	NS
Ewe mothering ability	1.26 (0.038)	1.32 (0.037)	1.33 (0.037)	NS

^*a*,*b*^Within a row, means without a common superscript differ (*P* ≤ 0.05).

NS, not significant (*P*>0.05).

### Lambing Performance

Ewe maternal genetic merit did not impact on lamb birth weight (*P* > 0.05, Table 2). Gestation length, which averaged 148.2 ± 0.17 d across the three genetic groups, did not differ by ewe maternal genetic merit (*P*>0.05, Table 2). Lambing difficulty scores were similar across the three groups (*P*>0.05, Table 2). Ewes of Irish origin, irrespective of maternal genetic merit, were more likely to suffer from dystocia (6.84 and 8.25 times for High and Low, respectively), compared with NZ ewes (*P*<0.001, Table 1) and averaged 12.7%, 24.0%, and 24.5% (raw means) for the NZ, High Irish, and Low Irish genetic groups, respectively.

Overall, lambs born from NZ ewes were 4.67 (95% CI: 1.89–11.55; *P*<0.001) and 6.54 (95% CI: 2.56–16.71; *P*<0.001) times more likely to successfully stand and suckle the ewe without assistance than lambs born from High Irish or Low Irish ewes, respectively (Table 1). Mothering ability did not differ by genetic group (*P*>0.05). The likelihood of lamb mortality occurring within the first 24 h postpartum did not differ between the three genetic groups (*P*>0.05).

### Productivity

**Figure 1.**
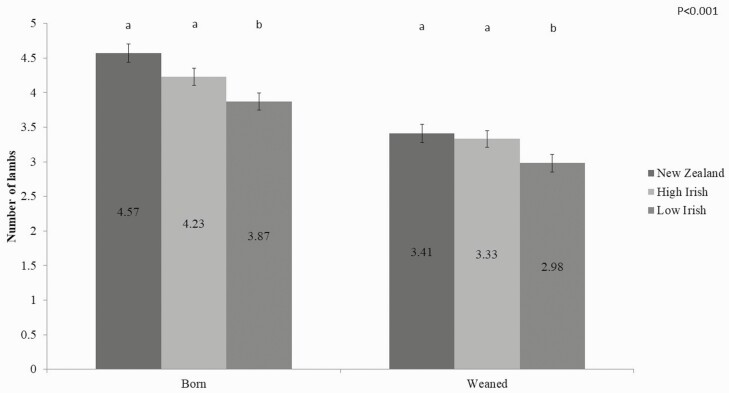
The effect of ewe maternal genetic merit (NZ, High Irish, Low Irish) on the total number of lambs recorded per ewe at birth (born) and at weaning (weaned), accumulated over the 4 yr of the study.

When the total number of lambs born per ewe over the 4 experimental years was combined, the NZ and High Irish ewes gave birth to more lambs per ewe when compared to the Low Irish group (*P* < 0.001, Figure 1). NZ and High Irish ewes also weaned a greater total number of lambs over the 4-yr study (3.41 and 3.33 lambs per ewe, respectively), compared with the Low Irish ewes (2.98 lambs per ewe; *P*<0.001, Figure 1).

## DISCUSSION

Genetics plays a substantial role in accelerating farm production gains. The benefit of using animals of high genetic merit has been quantified across beef (McHugh et al., 2014), dairy (Ramsbottom et al., 2012), and sheep (Marquez et al., 2013). For this reason, the authors hypothesized that animals of high genetic merit, whether of NZ or Irish origin, would achieve greater maternal performance than the animals of low genetic merit. However, results from the current study deviated from expectation, in that high genetic merit ewes of Irish origin did not always outperform their low Irish counterparts, albeit differences were detected in the total number of lambs born and weaned. The establishment of the Irish national sheep breeding program has initiated commercial farmers within the Irish sheep industry to seek change as they attempt to increase production gains, with an estimated 13% of breeding rams sourced from progressive pedigree breeders (Fetherstone et al., 2021). NZ has previously reported greater genetic gains relative to those achieved in Ireland (Santos et al., 2015), which prompted the comparison of high maternal genetic merit animals from NZ and Ireland within the same production system. This study helps to determine whether Irish genetics are lagging, surpassing, or reaching similar rates of genetic gain to those achievable through the use of NZ genetics.

Differences in reproductive efficiency including conception to first AI service and litter size are reported in this study. Farrell et al. (2020) discussed the impact of a high rate of ewe wastage on overall farm profitability in NZ, where ewe wastage was defined as ewes that were culled up to 6 yr of age or any mature ewe deaths. Ewe wastage is mainly driven by poor ewe reproductive performance (Cranston et al., 2017), and the importance of a minimal number of barren ewes within the flock at pregnancy scanning was demonstrated by Annett et al. (2011), who reported that they account for 40.8% of ewes culled per year, albeit in hill sheep flocks. Ewe barren rates did not differ between any of the genetic groups in this study (*P*>0.05) and were similar to the rates reported by Conington et al. (2004), although ewes within this study were mated via laparoscopic AI. Conception to first AI service rates within this study are in line with those reported previously by Hill et al. (1998) who also used fresh semen via laparoscopic AI and achieved an 82.2% conception rate; rates comparable to this study.

Number of lambs reared per ewe joined has been highlighted as a key driver of farm efficiency and profitability (Keady et al., 2009; Bohan et al., 2016; Farrell et al., 2020). Pregnancy scan rates and litter size observed in the current study were similar to those previously reported in Ireland (Bohan et al., 2016; Earle et al., 2017), but greater than those reported in previous NZ studies (DeNicolo et al., 2008; Morris and Kenyon, 2014), albeit across different breeds to those used in the present study.

Litter size increased by 23 lambs born per 100 NZ ewes lambed in comparison to the Low Irish ewes (*P*=0.05), which is not unsurprising given the greater response to selection in number of lambs born in the NZ maternal genetic index compared with the Irish maternal genetic index (Santos et al., 2015). These results demonstrate potential to increase these rates further within NZ where the national average for the number of lambs weaned per ewe joined is currently 1.30 lambs (Beef + Lamb New Zealand Economic Service, 2020) similar to that achieved on some of Ireland’s most profitable flocks (1.32 lambs per ewe joined) as reported by Kilcline et al. (2015).

Lambing difficulty, litter vigor, and ewe mothering ability have all been identified as the main contributors to the largest requirement of labor input during the lambing period (O’Brien, 2020). Although overall lambing difficulty scores did not differ across the three genetic groups, when transformed into a binary trait, i.e., dystocia, Irish ewes, regardless of maternal genetic merit, were more likely to suffer from dystocia than their NZ counterparts (*P<*0.001). This was partly attributed to the Low Irish ewes being 4.49 times more likely to suffer from lamb malpresentation at birth (*P*<0.001) and to the High Irish ewes being 4.09 times more likely to have oversized lambs (*P*<0.05), compared to the NZ ewes (results not shown). Overall lambing difficulty scores were considerably higher than expected in comparison to those reported previously by McHugh et al. (2020) and could be attributed to the differences in the breed composition used in both studies. Differences in lamb birth weight were not detected between the three genetic groups (*P*>0.05) and were greater than previously reported for Irish purebred lambs by McHugh et al. (2020) where raw means were presented as 5.12, 5.41, and 5.35 kg for NZ, High Irish, and Low Irish, respectively. The proportion of lambs that sucked independently was considerably less than that reported by Matheson et al. (2011), who allowed more time for lambs to stand and suckle before offering assistance when compared to the current study. The average percentage of litters that required assistance to suckle was 53.6%, 63.3%, and 67.1%, within the NZ, High Irish, and Low Irish groups, respectively. Differences in dystocia and litter vigor between NZ and Irish ewes may be attributed to the fact that the vast majority of producers in NZ operate an extensive, outdoor lambing system in which through natural selection, behavior may have adapted to result in a more easy-care system with minimal intervention at lambing (Fisher and Mellort, 2002). In comparison, Bohan et al. (2017) previously demonstrated the more intensive lambing system operated in Ireland whereby 83% of Irish producers lamb indoors. It should also be noted that birth weight is not included as a goal trait in either the Irish or NZ index. Maternal genetic merit had no impact on ewe mothering ability in this study, although scores were similar to those reported by Earle et al. (2017). The average perinatal lamb mortality rates were 7.2%, 8.2%, and 9.5% for NZ, High Irish, and Low Irish lambs, respectively, and did not differ between genetic groups but had a similar prevalance to those reported for single lambs by Morris et al. (2000).

Ewe survival is a major factor contributing to replacement costs. Currently, the average replacement rate in Ireland is 22.4% (Bohan et al., 2017), contributing to a high cost per kg of lamb produced on many Irish farms whereby the cost of a replacement ewe joining the flock at 18 mo is equivalent to 25% of the value of lamb carcass output that is produced in her lifetime (Keady, 2020). While replacement rate nationally in NZ ranges between 20% and 30%, a large proportion of these are recognized as annual ewe wastage, where premature death or culling of ewes prior to the end of their productive lifespan occurs (Farrell et al., 2020), albeit subjected to harsher environmental factors in late pregnancy, i.e., outdoor lambing, in comparison to a predominately indoor lambing system in Ireland. Unexpectedly, due to the higher replacement rates reported previously in NZ, ewes of NZ origin tended to survive longer in the flock as part of this study when compared to the Low Irish ewes (*P*=0.07), demonstrating their suitability in the Irish environment.

As previously mentioned, the number of lambs born is a key driver of performance and profitability. The significant focus on the number of lambs born within the Irish and NZ genetic indexes indicates that there is still potential to increase number of lambs born within the industry (Amer and Bodin, 2006; McHugh, 2016; Bohan et al., 2019). However, the ability of ewes to successfully rear their lambs (i.e., the difference between the number of lambs born and weaned) is not always reported. The artificial rearing, cross-fostering, and mortality of lambs between birth and weaning can impact profitability, increase production costs, and increase labor demand at a time of year when it is at a premium. While NZ ewes conceived and delivered the greatest amount of lambs on an annual basis, the total number of lambs born and weaned by NZ ewes was similar to the number achieved by High Irish ewes when analyzed over the period of the 4-yr study. As expected, NZ and High Irish ewes weaned a greater amount of lambs than the Low Irish ewes. Gottstein and Diskin (2020) highlighted that the Irish national annual weaning rate, at 1.3 lambs per ewe joined, has remained static for over 40 yr. The difference of 0.1 lambs per generation between ewes of high and low genetic merit in this study demonstrates that the national weaning rate could potentially be increased in a permanent and cumulative manner, through the use of superior genetics, regardless of country of origin, by generating replacement ewes of greater productivity potential.

Irish ewes within this study were selected for their divergence on the Irish maternal genetic index which incorporates a range of traits including: reproduction, lambing, growth, carcass and health traits with a different relative emphasis attributed to each trait (Bohan et al., 2019). Therefore, although it was hypothesized that Irish ewes of high genetic merit would achieve greater maternal performance than ewes of low genetic merit, an investigation of the individual Economic Breeding Value (EBV) for the various traits was required to illustrate whether differences in animal performance at an individual trait level were expected. Lamb survival and the number of lambs born per ewe have a large relative emphasis (8.98% and 18.19%, respectively) in the Irish maternal genetic index, compared to traits such as lambing difficulty [5.20% emphasis (Bohan et al., 2019)]. Therefore, it was anticipated that differences would be detected in animal performance for traits including lamb survival and the number of lambs born in comparison to lambing difficulty. Potentially greater differences could have been detected if more ewes were included within the study or the study was repeated over a larger number of years. Differences between the individual trait EBVs of Irish ewes of high and low genetic merit and the actual phenotypic divergence between the corresponding traits in this study were broadly as expected. For example, the difference at the EBV level for the number of lambs born between the High and Low Irish genetic groups was 0.04; the corresponding phenotypic difference observed in this study was 0.03 lambs born per ewe. In fact, based on the individual EBVs, greater divergence is anticipated based on growth traits such as weaning and slaughter weight between the Irish High and Low groups rather than for the maternal traits investigated in the present study. Other performance traits such as ewe survival, lamb birth weight, and ewe mothering ability reported within this study are currently not included in the Irish maternal genetic index; therefore, overall performance and EBVs cannot be compared at this time. In addition, it should be highlighted that the accuracy levels associated with the Irish maternal genetic index are still relatively low (ranged from 34% to 43% for the three genetic groups), which can result in changes to the overall indexes and ranking of animals. In fact, over the course of the study, only 26% and 52% of ewes remained ranked within the top and bottom 20% for maternal genetic merit, respectively. This could in part be attributed to continuous development of the Irish maternal genetic index including the incorporation of new traits, namely, dagginess and lameness (O’Brien et al., 2017) and the introduction of across breed genomic evaluations (Pabiou et al., 2019).

## CONCLUSION

Results from this study provide a detailed comparison of the reproductive, lambing, and productivity for ewes of NZ and Irish origin and of high and low maternal genetic merit, which can be used in order to increase subsequent on-farm productivity through educated decision-making. Results demonstrate the suitability of NZ genetics within an Irish production system and show NZ ewes to be at least similar, if not superior, to their High Irish counterparts for all traits recorded. NZ ewes had superior reproductive and lambing performance for many traits including litter size, conception to first AI service, dystocia, and litter vigor, when compared to low maternal genetic merit animals. Although differences between the High Irish and Low Irish ewes were not evident within many traits, the number of lambs born and weaned when accumulated over the 4 experimental years demonstrated the benefit of selecting toward animals of high maternal genetic merit over time and the potential for both farmers and the national sheep industry as a whole to increase output via the number of lambs produced and traded each year.

## DATA AND MODEL AVAILABILITY STATEMENT

The data and models reported within this study are available from the corresponding author upon reasonable request.
